# The Novel Atypical Antipsychotic Lurasidone Affects Cytochrome P450 Expression in the Liver and Peripheral Blood Lymphocytes

**DOI:** 10.3390/ijms242316796

**Published:** 2023-11-27

**Authors:** Przemysław J. Danek, Władysława A. Daniel

**Affiliations:** Department of Pharmacokinetics and Drug Metabolism, Maj Institute of Pharmacology, Polish Academy of Sciences, Smętna 12, 31-343 Kraków, Poland; danek@if-pan.krakow.pl

**Keywords:** lurasidone, chronic treatment, liver, blood lymphocytes, cytochrome P450 expression

## Abstract

Lurasidone is a novel atypical antipsychotic drug acting on dopaminergic, serotonergic and noradrenergic receptors; it is applied for the long-term treatment of schizophrenia and depression in patients with bipolar disorders. We aimed at performing a comparative study on the influence of chronic treatment with lurasidone on the expression of cytochrome P450 enzymes in the liver and in peripheral blood lymphocytes, and to evaluate the relationship between changes in the expression of CYP enzymes in the two experimental models. The obtained results show a fairly similar expression pattern of the main CYP enzymes in the rat livers and lymphocytes, and they indicate that in the liver, lurasidone exerts an inhibitory effect on the activity, protein and mRNA levels of CYP2B1/2 (not *CYP2B2* mRNA), CYP2C11 and CYP2E1, while in the case of CYP3A1 and CYP3A2, it causes enzyme induction. At the same time, lurasidone decreases the expression of CYP2B, CYP2C11 (CYP2C11 protein only) and CYP2E1 but increases that of CYP3A2 (not CYP3A1) in lymphocyte cells. In conclusion, chronic treatment with lurasidone simultaneously and in the same way influences the expression and activity of CYP2B, CYP2C11, CYP2E1 and CYP3A2 in the liver and peripheral blood lymphocytes of rats. Thus, the lymphocyte cytochrome P450 profile may be utilized as an indicator of the hepatic cytochrome P450 profile in further clinical studies with lurasidone, and lymphocytes may serve as easily available surrogates for examining the impact of new drugs and chronic in vivo treatments on CYP enzyme expression, as well as to estimate drug–drug interactions and toxicity risk.

## 1. Introduction

Cytochromes P450 (CYPs) are a superfamily of hemoproteins involved in the oxidative metabolism of xenobiotics, including psychotropics, environmental pollutants and drugs. CYPs also catalyze the metabolism of endogenous compounds, such as steroid hormones, eicosanoids, vitamins, arachidonic acid and bile acids [1]. Cytochrome P450, due to the abundance of its forms and the broad spectrum of its catalytic activities, is an enzyme of great physiological, pharmacological and toxicological importance. CYPs are chiefly located in the liver; however, they exist in almost every organ/tissue found in the brain [2], lungs [3], and kidneys [4], as well as in lymphocytes [5].

For many years, the catalytic competencies and regulatory mechanisms of CYPs occurring in the liver have been the subject of intense research, which has provided valuable scientific and medical information. Until now, research has focused mainly on the regulation of cytochrome P450 expression in the liver; however, the latest data indicate a variable expression of CYPs in peripheral blood lymphocytes (PBL); thus, they may be used as accessible surrogate cells for CYP enzyme expression studies [6]. It was demonstrated that CYPs expressed in PBL were functioning and showed a similar pattern of regulation as in the liver [7]. Lymphocytes have been recognized as an appropriate cell model to investigate the expression of rat enzymes belonging to the subfamilies CYP1A, CYP2A, CYP2B, CYP2E and CYP3A [8,9,10,11,12,13] and the human enzymes CYP2E1, CYP1A1, CYP1A2, CYP2C9, CYP2C19 and CYP3A4 [5,14,15,16,17]. Moreover, the expression of CYP enzymes along with corresponding transcription factors and transporters has also been documented in PBL, suggesting that PBL could be used in a diversity of clinical studies [18]. However, some contradictory results have been reported regarding certain human CYPs concerning the correlation between the inducibility level of human CYP1A1 and CYP1B1 in PBL and the liver [19]; the correlation between the expression of CYP1A2, CYP2C19, CYP2D6 and CYP3A4 in PBL and the systemic activity of these enzymes in human subjects treated with the wide-spectrum inducer rifampicin [20]; and the correlation between the expression of CYP2B6 and CYP2D6 in PBL and the hepatic activities of these enzymes [17].

Since therapies with psychotropic drugs (e.g., antipsychotics and antidepressants) are long-lasting and new psychotropics are being systematically introduced, there is a need to apply an experimental model that can easily demonstrate the effects of prolonged administration of a new drug on the regulation of liver cytochrome P450 in psychiatric patients. The existing in vitro models (e.g., hepatocyte culture) cannot show the full-spectrum effects of a centrally active drug on liver CYPs, since some of them may be regulated by neuroendocrine mechanisms [21]. Therefore, finding the correlations between the CYP enzyme regulation in hepatocytes and accessible blood lymphocytes from patients could enable the observation of changes in CYP enzyme expression during long-term pharmacotherapy, as well as their correlation with CYP metabolic function in the liver.

Our recent studies showed that lurasidone directly inhibited the enzymatic activity of cytochrome P450 protein in vitro in human liver microsomes [22] and did not affect CYP enzyme expression in human hepatocyte cells [23]; meanwhile, chronic treatment of rats with the antipsychotic affected the expression and activity of CYP2B, CYP2C11 and CYP3A in the liver [24]. Lurasidone is a novel second-generation antipsychotic drug used for the treatment of schizophrenia and depression in patients with bipolar disorders; it acts as an antagonist of dopamine D2 and serotonin 5-HT2A and 5-HT7 receptors, a partial agonist for 5-HT1A receptors, and a moderate ligand for α2A and α2C adrenergic receptors [25,26]. Lurasidone belongs to the benzisothiazole derivative class, which is mainly bio-transformed in the human liver by CYP3A4 into three active metabolites (ID-14283, ID-14326 and ID-14614) and two inactive metabolites (ID-20219 and its hydroxylated derivative ID-20220) [27,28].

Pharmacotherapies of psychiatric disorders are conducted for months/years to achieve and maintain therapeutic effect, and to prevent relapses. Considering the previously observed effects of lurasidone on cytochrome P450 expression in rat livers, we chose this antipsychotic drug for a comparative study on the effect of chronic treatment on the expression of CYP enzymes (including CYP1A, CYP2B, CYP2C11, CYP3A and CYP2E1) in the liver and in peripheral blood lymphocytes, in order to evaluate the correlation between alterations in the expression of CYP enzymes in those two experimental models.

## 2. Results

### 2.1. CYP Enzyme Activity in Rat Liver Microsomes after Prolonged Lurasidone Administration

**Figure 1:**
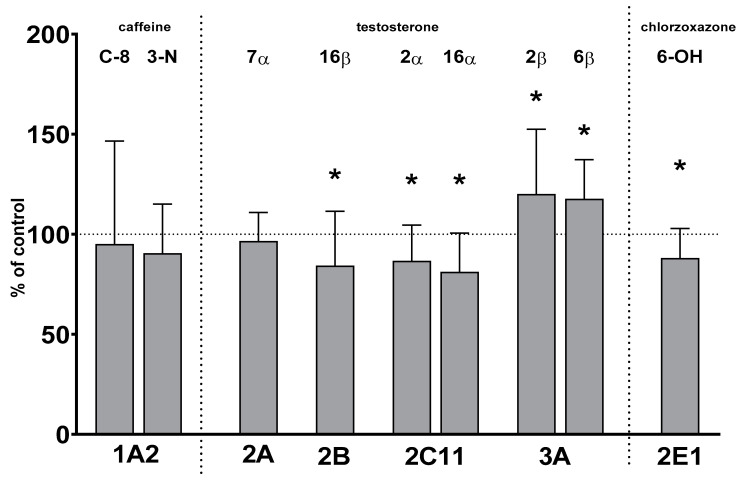
The effect of chronic treatment with lurasidone on the activity of cytochrome P450 enzymes in liver microsomes. The activities of CYP enzymes were measured as the rates of CYP-specific reactions: caffeine 8-hydroxylation and 3-N-demethylation (CYP1A), testosterone 7α- (CYP2A), 16β- (CYP2B), 2α- and 16α- (CYP2C11), and 2β- and 6β- (CYP3A) hydroxylation, and chlorzoxazone 6-hydroxylation (CYP2E1). All results are presented as the mean ± SD (n = 15 rats). Student’s *t*-test: * *p* < 0.05 versus respective control. The control values are as follows: 8.73 ± 2.76 pmol/mg protein/min (caffeine 8-hydroxylation); 0.82 ± 0.28 pmol/mg protein/min (caffeine 3-N-demethylation); 211 ± 29 pmol/mg protein/min, 119 ± 38 pmol/mg protein/min, 1.392 ± 0.41 nmol/mg protein/min, 1.605 ± 0.45 nmol/mg protein/min, 136 ± 21 pmol/mg protein/min and 1.297 ± 0.2 nmol/mg protein/min (testosterone 7α-, 16β-, 2α-, 16α-, 2β- and 6β-hydroxylation, respectively); and 2.84 ± 0.35 nmol/mg protein/min (chlorzoxazone 6-hydroxylation).

Two-week treatment with lurasidone significantly reduced the activity of CYP2B, CYP2C11 and CYP2E1 but increased that of CYP3A. The activity of CYP1A and CYP2A was not affected by lurasidone (Figure 1).

The activity of CYP2B decreased to 84% of the control value. The activity of CYP2C11 declined to 86% and 81% of that of the control, respectively. The activity of CYP2E1 fell to 88% of that of the control. In contrast, the activity of CYP3A increased to 120% and 117% of that of the control, respectively. The activity of CYP1A and CYP2A remained unchanged after lurasidone administration (Figure 1).

### 2.2. CYP Protein Level in Liver Microsomes and Lymphocytes after Chronic Lurasidone Treatment

**Figure 2:**
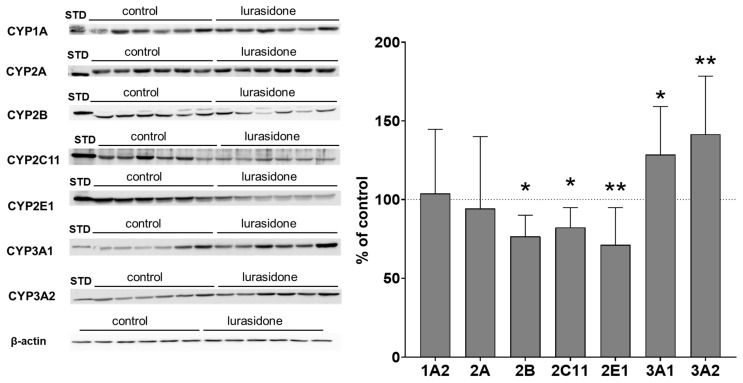
The influence of prolonged treatment with lurasidone on the protein level of CYP1A, CYP2A, CYP2B, CYP2C11, CYP2E1, CYP3A1 and CYP3A2 in the rat liver microsomes. Microsomes (10 μg protein per lane) were analyzed by Western immunoblotting. The results are demonstrated as representative blots from six (for the control and for lurasidone treatment) rats per group. The data are shown as the mean ± SD (n = 12 rats). Student’s *t*-test: * *p* < 0.05, ** *p* < 0.01 versus respective control. STD: standard.

The observed alterations in CYP protein levels were consistent with the changes in CYP activity in liver microsomes. The CYP2B protein level was reduced to 76% of that of the control value. Lurasidone significantly diminished the CYP2C11 protein level to 82% of that of the control. The protein level of CYP2E1 was decreased by the tested antipsychotic to 71% of that of the control. The CYP3A1 and CYP3A2 protein levels were significantly increased by lurasidone to 128% and 141% of that of the control value, respectively. Lurasidone did not affect the CYP1A2 and CYP2A protein levels (Figure 2, Supplemental Data).

**Figure 3:**
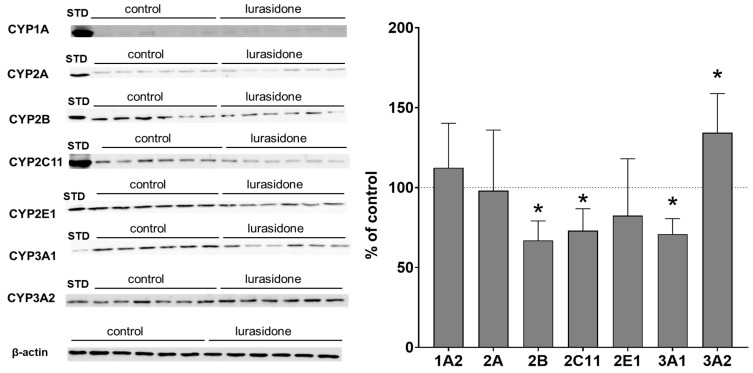
The influence of chronic treatment with lurasidone on the protein levels of CYP1A, CYP2A, CYP2B, CYP2C11, CYP2E1, CYP3A1 and CYP3A2 in the rat lymphocytes. Lymphocytes (50 μg protein, derived from 2 rats, 1:1) were analyzed by Western blotting. The results are demonstrated as representative blots from six (for the control and for lurasidone treatment) rats per group. The data are shown as the mean ± SD (n = 6 samples, each from 2 animals). Student’s *t*-test: * *p* < 0.05 versus respective control. STD, standard.

The investigated CYP enzyme proteins were present in PBL but at distinctly lower levels than in the liver, as indicated in the Western blot analysis images. The protein level used for lymphocytes was five-fold higher and the secondary antibody concentration was four-fold higher than that used for liver microsomes. Despite the modifications used, Western blot analyses showed much lower values than those observed for liver microsomes, as indicated in Figure 2 and Figure 3. Similarly to the liver microsomes, Western blot analysis revealed changes in the examined CYP enzyme proteins in PBL isolated from rats treated with lurasidone. The observed alterations were similar in lymphocytes compared to liver microsomes. Lurasidone significantly reduced CYP2B, 2C11 and CYP3A1 protein levels to 66%, 72% and 70% of that of the control, respectively, but increased the CYP3A2 protein level to 134% of that of the control. The CYP1A2 and CYP2A protein levels were not changed by lurasidone. The antipsychotic tended to decrease the CYP2E1 protein level (Figure 3, Supplemental Data).

### 2.3. Alterations in the CYP mRNA Levels in the Liver and Lymphocyte Cells after Lurasidone Treatment

**Figure 4:**
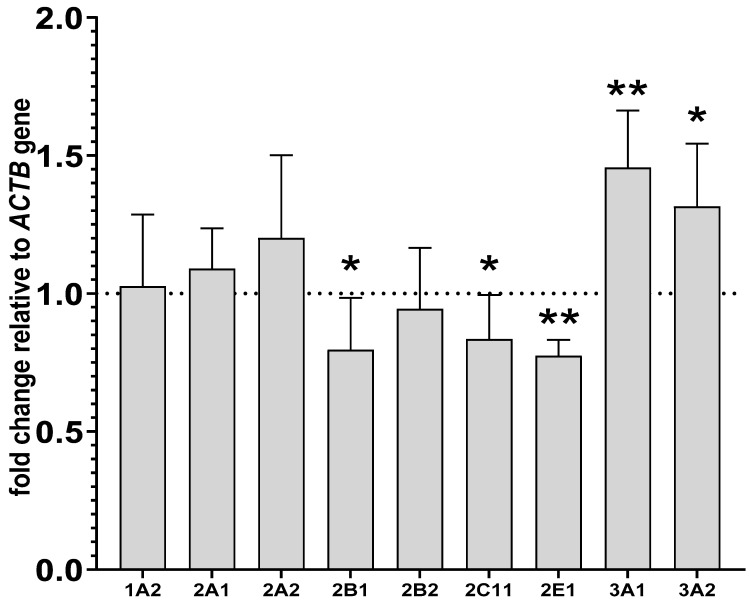
The influence of two-week treatment with lurasidone on the mRNA levels of *CYP1A2*, *CYP2A1*, *CYP2A2*, *CYP2B1*, *CYP2B2*, *CYP2C11*, *CYP3A1*, *CYP3A2* and *CYP2E1* genes in the liver. The results are expressed as the fold-change in relation to the reference gene ACTB. All the values denote the mean fold-change calculated by the comparative delta-delta Ct method for the control and lurasidone-treated rats. All values are expressed as means ± SD (n = 10 rats). Student’s *t*-test: * *p* < 0.05, ** *p* < 0.01 compared to the control group.

The expression of ACTB, a reference gene, was most stable in all tested samples. The altered activities and protein levels of CYP2B, CYP2C11 and CYP3A in the liver remained in agreement with changes in the CYP mRNA levels found after prolonged drug administration. Lurasidone reduced the levels of CYP2B1, CYP2C11 and CYP2E1 mRNAs to 79%, 83% and 77% of that of the control, respectively. The tested antipsychotic increased the CYP3A1 and CYP3A2 mRNA levels to 145% and 131% of that of the control, respectively. Lurasidone did not cause any significant effects in the mRNA levels of CYP1A2, CYP2A1 and CYP2A2 (Figure 4).

**Figure 5:**
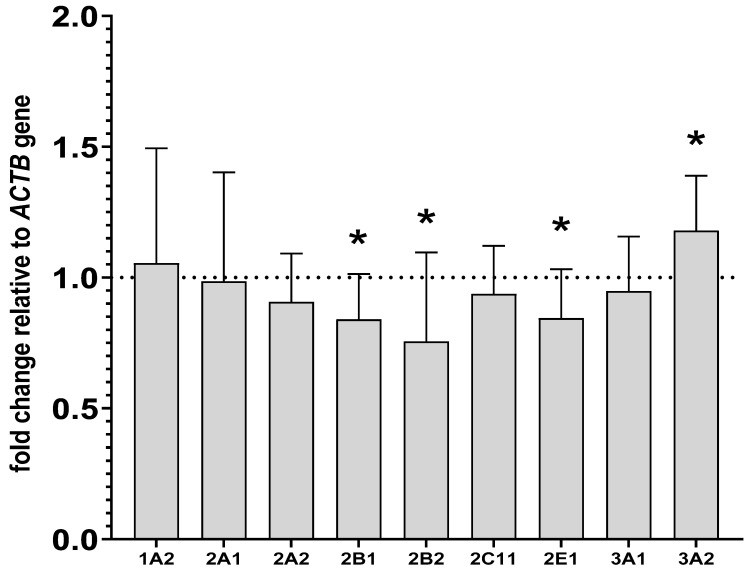
The influence of two-week treatment with lurasidone on the mRNA levels of *CYP1A2*, *CYP2A1*, *CYP2A2*, *CYP2B1*, *CYP2B2*, *CYP2C11*, *CYP3A1*, *CYP3A2* and *CYP2E1* genes in the lymphocyte cells. The results are expressed as the fold-change relative to the reference gene ACTB. All values denote the mean fold-change calculated by the comparative delta-delta Ct method for the control and lurasidone-treated rats. All values are expressed as means ± SD (n = 8 rats). Student’s *t*-test: * *p* < 0.05 compared to the control group.

The investigated CYP genes were found to be expressed in PBL, but quantification studies revealed that CYP genes in PBL were expressed at lower levels than in the liver, as indicated by much higher cycle levels in the RT-PCR analysis for livers. For example, for the CYP2B1 gene in the liver, the cycle level was approximately 20; meanwhile, for the same gene in lymphocytes, the number of cycles was 30. In the isolated lymphocytes, lurasidone decreased the mRNA levels of CYP2B1 and CYP2B2 to 83% and 75% of that of the control, respectively. Similarly to the liver, lurasidone reduced the CYP2E1 mRNA level to 84% of that of the control and raised the CYP3A2 mRNA level to 117% of that of the control in lymphocytes. However, in contrast to the liver, lurasidone did not influence significantly the CYP2C11 and CYP3A1 mRNAs. As in the liver, the CYP1A2, CYP2A1 and CYP2A2 mRNAs were not altered after lurasidone administration (Figure 5).

## 3. Discussion

This is the first study to examine the parallel expression and activity of CYP enzymes in rat PBL and livers after chronic lurasidone administration. The presented data show a fairly similar pattern of expression of the main rat CYP enzymes in the liver and blood lymphocyte cells, though some variances can be noticed (Table 1). The data indicate that lurasidone exerts an inhibitory effect on the activity, protein and mRNA levels of *CYP2B1/2* (not *CYP2B2* mRNA), CYP2C11 and CYP2E1, while in the case of CYP3A1 and CYP3A2, it causes enzyme induction in rat livers. At the same time, lurasidone decreases the expression of CYP2B, CYP2C11 (CYP2C11 protein only) and CYP2E1 but increases that of CYP3A2 (not CYP3A1) in rat peripheral lymphocyte cells. Certain differences in lurasidone effects on CYP enzyme expression (concerning CYP2B2, CYP2C11 and CYP3A1) observed between the liver and lymphocytes may be associated with differences in the expression levels of transcription factors, drug and metabolite concentrations within hepatocytes and lymphocytes, and the presence of neurotransmitter and hormone receptors on/in those cells [21,29].

As mentioned before, lurasidone can influence intracellular signaling via dopaminergic, serotonergic and adrenergic receptors [25,26]. Moreover, the drug can in this way affect hormone secretion via neuroendocrine mechanisms [21]. Growth hormone is the main regulator of rat CYP2C11, while corticosterone is the main regulator of CYP3A enzymes and contributes to the regulation of other CYP enzymes [29]. Therefore, changes in neurotransmitter and hormonal signaling produced by lurasidone might influence CYP enzyme expression in a tissue/cell-dependent manner.

In general, the results of our present work obtained after two-week intraperitoneal treatment are similar to those obtained in our previous study performed after five-week per os treatment of rats with lurasidone [24], though certain differences can be seen. In both cases, a decrease in the CYP2B enzyme activity and protein level but no change in the CYP2B2 mRNA level was observed in the liver. However, the CYP2B1 mRNA was decreased after two-week treatment, whereas it increased after five-week treatment with lurasidone. On the other hand, the expression of CYP3A1/2 was elevated after two-week and five-week treatment with the antipsychotic; however, the enzyme activity was enhanced after two-week treatment only. The observed time-dependent effects of lurasidone on CYP2B1 expression and CYP3A activity may be connected with different levels of drug metabolites accumulating in the liver after two-week intraperitoneal treatment and five-week per os treatment, which can affect *CYP* gene transcription or CYP protein activity.

Drug–drug interactions may occur as a consequence of induction or inhibition of the expression of drug metabolizing enzymes, or as a result of the direct blocking of enzyme protein. A few scientific reports suggest that antipsychotic drugs can induce cytochrome P450 in rat liver. The induction of CYP3A by clozapine and risperidone [30,31] and CYP2E1 induction by iloperidone [32] have been reported. In this study, lurasidone enhanced the activity, protein and mRNA level of *CYP3A1/2* in rat livers, and a similar effect was observed in lymphocytes for CYP3A2. Thus, in the case of lurasidone, both direct and indirect mechanisms seem to be engaged in the regulation of cytochrome P450 activity in the liver: direct inhibition of CYP enzyme proteins, as shown in human liver microsomes (inhibition of CYP1A2, CYP2C9/19, CYP2D6 and CYP3A4) [22] and the indirect effect on *CYP* gene expression (e.g., CYP3A1/2 enzyme induction) [24], as shown in the present work in vivo. The final effect of lurasidone on the activity of a particular CYP enzyme in vivo is the result of both mechanisms and depends on the dose, treatment duration and time interval after the last dose. Since lurasidone is administered to psychiatric patients for a long time and also to individuals who may be treated simultaneously with other drugs/substrates of CYP3A (e.g., erythromycin, midazolam, carbamazepine, immunosuppressive drugs, calcium channel blockers and corticosteroids), it may influence the biotransformation of the co-medications and their pharmacological effects [1]. Moreover, changes in hepatic CYP3A activity via lurasidone may affect the metabolism of important endogenous substances (e.g., steroids), contributing to serious side-effects [33]. In addition, lurasidone may produce autoinduction, since the drug is metabolized mainly by CYP3A, and in our study this antipsychotic induced the expression and activity of this enzyme. On the other hand, our results show that lurasidone exerts an inhibitory effect on the expression of CYP2B, CYP2C11 and CYP2E1.

These results accord with our earlier study, where other second-generation neuroleptics (asenapine and iloperidone) were found to decrease the expression and activity of CYP2B and CYP2C11 [32,34]; this suggests the involvement of pharmacological receptors. As already mentioned, the observed decreases in CYP enzyme expression and activity elicited by new atypical neuroleptics may be associated with the neuroendocrine mechanisms. Brain monoaminergic systems (noradrenergic, dopaminergic and serotoninergic) are engaged in the regulation of CYP enzymes in the liver [21]. These neurotransmitter systems regulate the concentration of hormones in the plasma, including corticosterone, growth hormones and thyroid hormones, which regulate cytochrome P450 expression and activity [21,29]. By acting at monoaminergic neurotransmitter receptors in the hypothalamic nuclei and pituitary, atypical antipsychotics, including lurasidone (an antagonist of D_2_, 5-HT_2A_, 5-HT_7_, α_2A_, α_2C_ and a partial agonist of 5-HT_1A_ receptors) may influence hormone secretion and, in turn, CYP enzyme expression. No changes in cytochrome P450 expression or activity were observed in hepatocyte culture after a five-day incubation with lurasidone in vitro [23], which supports the abovementioned hypothetical pharmacological mechanism of enzyme regulation by neuroactive drugs in vivo.

As mentioned elsewhere, previous studies have also indicated similarities in the regulation of expression of CYP enzymes in rats’ livers and lymphocyte cells. Those studies showed the same pattern of expression and regulation of CYP1A1 and CYP1A2 in the rat liver and lymphocytes after pretreatment with 3-methylcholanthrene [10,12]. Positive correlations between the liver and PBL in the CYP2B1/2B2 regulation (including the enzyme activity, protein and mRNA levels) were found after pretreatment of rats with phenobarbital [6]. Dey et al. [8] demonstrated similarities in the regulation and catalytic activity of CYP3A enzymes in rat blood lymphocytes and in the liver after pretreatment of rats with dexamethasone. Significant increases in the expression and regulation of CYP2A enzymes were found in in the PBL of male and female rats pretreated with nicotine or 3-methylcholanthrene, and in the PBL derived from lung cancer patients [13]. Moreover, it was shown that the levels of CYP1A and CYP2E1 were increased in the lymphocytes of patients suffering from tobacco-induced lung cancer and alcoholic liver diseases, respectively [15,16,35]. Lin et al. [36] reported the induction of CYP1A1 and CYP1B1 in human lymphocytes from males and females evoked by smoking or by the incubating of lymphocytes with benzanthracene in vitro. Lampe et al. [37] observed an elevation in CYP1B1 in the leukocytes of smokers and a significant correlation with plasma cotinine. In another study, Hannon-Fletcher and Barnett [11] showed an enhancement in the expression of the CYP2B, CYP2E and CYP3A in the lymphocytes of rats pretreated with specific CYP inducers. The alterations in transcriptional factors (CAR, PXR, AhR, PPAR, RXR and LXR) engaged in the regulation of CYP enzymes have shown similarities in the expression mechanisms of CYP enzymes in PBL and in the livers of rats and humans [7,38,39]. However, in every study, the magnitude of expression of CYP enzymes was higher in the liver compared to the lymphocyte cells, which was also observed in our experiment.

In summary, the results of the present study documented similarities in the expression of CYP enzymes between the liver and PBL after lurasidone treatment. Similarly to the liver, in rat peripheral lymphocyte cells, lurasidone decreased the expression of CYP2B, CYP2C11 and CYP2E1 but increased the expression of CYP3A2. Moreover, Western blot and RT-PCR studies demonstrated a markedly lower level of CYP enzymes in the lymphocytes of control and lurasidone-treated rats than those observed in liver microsomes. Nonetheless, the direction of changes in protein and mRNA levels was the same (except for CYP3A1) as observed in the liver. It seems that in rat lymphocytes, CYP3A2 but not CYP3A1 is indicative of liver CYP3A function under lurasidone treatment.

However, it still remains to be elucidated whether CYP3A1 or CYP3A2 better reflects human CYP3A4 under neuroleptic treatment. In general, the regulation of hepatic CYP3A enzymes differs between rats and humans (reviewed in [29,40]). Rifampicin is a strong inducer of human CYP3A4 (but not rat CYP3A), while dexamethasone is a potent inducer of rat CYP3A and a weak inducer of the human enzyme. These differences in enzyme induction may be due to the different efficacies of inducers to activate nuclear receptors involved in CYP3A gene expression in rodents and humans. On the other hand, in rat livers, dexamethasone induced CYP3A1 with a greater potency than CYP3A2, asenapine induced only CYP3A1, and zolmitriptan stimulated solely CYP3A2 [34,41], which indicates different mechanisms of regulation for CYP3A1/2 enzymes. In addition, the expression levels of nuclear receptors/transcription factors may differ between lymphocytes and hepatocytes, which can lead to the differential response of CYP3A enzymes to lurasidone in those cells.

## 4. Materials and Methods

### 4.1. Animals

Male Wistar Han rats (Charles River Laboratory, Sulzfeld, Germany) at the age of 3 months and weighing approximately 270–300 g were housed in a standard cage (temperature 22 ± 2 °C, humidity 50 ± 5% and 12:12 light/dark cycle) with free access to laboratory water and food. All studies were carried out using procedures ethically approved by the Local Ethics Commission for Experimentation on Animals at the Maj Institute of Pharmacology, Polish Academy of Sciences, Kraków, and in compliance with relevant guidelines and regulations of the 2010/63/EU Directive and the Guide for the Care and Use of Laboratory Animals.

### 4.2. Animal Treatment

Lurasidone (1 mg/kg/day) (TargetMol, Boston, MA, USA) dissolved in 0.5% methylcellulose and 0.2% Tween 80 in water was administrated intraperitoneally. The dose was selected on the basis of pharmacological, neurochemical and behavioral paradigms demonstrating antipsychotic properties, as established in previous studies [42,43,44]. Rats (n = 15 for the control and drug treatment group) received drug or vehicle once a day over 2 weeks [24]. Based on our previous experiments, a period of 2 weeks is sufficient to observe drug-induced changes over time [32,34]. Then, rats were decapitated 24 h after the final dose and blood samples (approx. 8 mL) were collected into tubes containing ethylenediaminetetraacetic acid (EDTA). Whole livers were removed, frozen in dry ice and then stored at −80 °C until further analysis.

### 4.3. Estimation of CYP Enzyme Activities in Liver Microsomes

Microsomes were obtained from separate livers in accordance with a previously described method [31]. Briefly, livers were thawed on ice, fragmented into small pieces and homogenized in 4 vol. of 20 mM Tris/KCl buffer (pH = 7.4). Then, homogenates were centrifuged at 10,000× *g*, and supernatants were centrifuged at 100,000× *g* for 1 h. Next, microsomes were washed with 0.15 mM KCl, and centrifuged at 100,000× *g* for 1 h. The prepared microsomal pellet was then suspended in 20 mM Tris/KCl buffer, pH 7.4 with 20% (*v*/*v*) sucrose to yield a concentration of 20 mg protein/mL; the suspension was stored at −80 °C.

Cytochrome P450 enzyme activity in rat liver microsomes was measured using CYP-specific reactions according to the previously described methods [36]. Briefly, the activity of CYP1A was measured as the rate of caffeine C-8-hydroxylation and 3-N-demetylation at a substrate concentration of 100 μM (Sigma, St. Louis, MO, USA), the activity of CYP2E1 was measured as the rate of 6-hydroxylation of chlorzoxazone at a substrate concentration of 200 μM (Sigma, St. Louis, MO, USA), while the activities of CYP2A, CYP2B, CYP2C11 and CYP3A were estimated by measuring the rates of the 7α-, 16β-, 2α- and 16α-, and 2β- and 6β-hydroxylation of testosterone, respectively, at a substrate concentration of 100 μM (Steraloids, NewPort, KY, USA) [31,45]. The above probe reactions for the control and lurasidone treatment groups were performed in an incubation system containing 50 mM TRIS/KCL buffer, 5 mM glucose 6-phosphate, 1.7 U/mL glucose 6-phosphate dehydrogenase, 1 mM NADP or 1 mM NADPH with 0.15 M phosphate buffer for CYP1A2 reaction (Sigma, St. Louis, MO, USA). All reactions were assessed by the HPLC method via UV detection.

### 4.4. Lymphocyte Isolation

The investigated cell samples contained mainly lymphocytes (>80%), with considerably fewer monocytes, neutrophils, granulocytes, erythrocytes and platelets. The isolated cells may be called peripheral blood mononuclear cells. However, since most of the cells are lymphocytes, these samples are generally referred to as peripheral blood lymphocytes (PBL) for ease of nomenclature, as reflected in the scientific literature [12]. Lymphocytes were isolated from whole rat blood based on the method described by Böyum [46], with some modifications. In brief, whole blood was diluted 1:1 with phosphate-buffered saline (PBS, pH = 7.4) and carefully layered on Ficoll-Paque Plus (Sigma, St. Louis, MO, USA) according to the following proportion: 0.65 mL Ficoll-Paque Plus per 1 mL of diluted blood. Then, the diluted blood was centrifuged at 700× *g* for 30 min at room temperature. The upper layer was discarded and the lymphocyte cells were aspirated carefully, washed twice using PBS buffer and resuspended in 0.5 mL PBS. The lymphocytes samples were stored at −80 °C.

### 4.5. Western Blotting

Because of the small amounts of microsomal proteins in the isolated lymphocytes, the cells did not undergo the procedure of microsome preparation and CYP activity determination but were used straight away for the analyses of CYP enzyme expression (CYP protein and CYP mRNA measurement). Lymphocytes were homogenized by sonication in 20 volumes (*v*/*v*) of PBS buffer and centrifuged at 15,000× *g* for 15 min at 4 °C. The estimation of total protein in lymphocytes and liver microsomes was performed using a Pierce BCA Protein Assay Kit (ThermoFisher, Walthman, MA, USA). Western immunoblot analysis was carried out according to the previously described method [32]. Briefly, microsomal proteins (10 μg) or lymphocyte proteins (50 μg) were separated on SDS (12%) gels in a dilution of Laemmli buffer (Bio-Rad, Hercules, CA, USA). Appropriate cDNA-expressed rat CYP Supersomes were applied as a standard/positive control (Gentest Corp., Woburn, MA, USA) [32]. Afterwards, protein samples were transferred onto a nitrocellulose membrane (Amersham Protran, Merck KGaA, Darmstadt, Germany). The membranes were probed with primary antibodies: anti-rat CYP1A1/2, CYP3A1, and CYP3A2 antibody obtained from Millipore (Temecula, CA, USA); anti-rat CYP2C11 and CYP2E1 antibody obtained from (Thermo Fisher Scientific, Walthman, MA, USA); and monoclonal mouse anti-rat CYP2B1/2B2 antibody purchased from Santa Cruz Biotechnology (Dallas, TX, USA). Polyclonal anti-rat β-actin antibody was supplied by Sigma (St. Louis, MO, USA) (Table 2). All antibodies were diluted in SignalBoostTMImmunoreaction Enhancer Kit (Millipore, Burlington, MA, USA) according to the previously described method [32], and the bands were imaged via extended chemiluminescence signals. The immunoblots were analyzed using a luminescent image analyzer and professional software (Image Gauge V4.0 and LAS-1000, Fuji-film, Tokio, Japan). The β-actin signal was used for normalization of the obtained data.

### 4.6. Assay for CYP mRNA Expression

Total RNA Mini kit (A&A Biotechnology, Gdynia, Poland) was used for isolation of the RNA from frozen liver or lymphocyte cells following the producer’s protocol. The quality and concentration of the RNA were estimated spectrophotometrically by a Synergy/HTX multi-mode reader (BioTek, Winoosk, VT, USA), and A260/280 ratios were between 1.8 and 2.0 in all the tested samples. Reverse transcription was conducted using a High Capacity cDNA Reverse Transcription Kit (Life Technologies, Carlsbad, CA, USA) in agreement with the producer’s protocol. Real-time PCR analysis was conducted in duplicate with TaqMan type probes and primers, TaqMan Expression Master Mix (Life Technologies, Carlsbad, CA, USA) [32] and Bio-Rad CFX96 PCR system (Bio-Rad, Hercules, CA, USA). The housekeeping genes (ACTB, GAPDH and HPRT) were quantified to references. The abundance of RNA was calculated as 2-delta Ct, as described previously [32].

### 4.7. Statistical Analysis

The Kolmogorov–Smirnov test was used to assess normality. Changes in the activity, protein and gene expression levels of CYP enzymes evoked by lurasidone were evaluated for statistical significance using the Student’s *t*-test (GraphPad Prism 9.5.1 Software Inc., San Diego, CA, USA). The obtained results were presented in graphs as the mean values with ± SD and were regarded as statically significant when *p* < 0.05.

## 5. Conclusions

The present study provides evidence that chronic treatment with lurasidone simultaneously and in the same way influences the expression and activity of CYP2B, CYP2C11, CYP2E1 and CYP3A2 in the liver and peripheral blood lymphocytes. Considering some similarities in the regulation and catalytic competence between the tested rat and human CYPs, pharmacokinetic interactions at the level of CYP-mediated metabolism can be expected in lurasidone-treated patients. Pharmacokinetic interactions involving lurasidone and substrates of CYP2B6 (lidocaine, bupropion and selegiline), CYP2C9 (warfarin and diclofenac), CYP2E1 (ethanol and isoflurane) or CYP3A4 (statins, benzodiazepines and antibiotics) are likely to occur in patients during combined therapy [1]. Such interactions may have medical implications, which should be taken into consideration by physicians during combined pharmacotherapy.

Hepatic cytochrome P450 enzymes are considered to best characterize drug biotransformation in the body; however, CYP expression in PBL may provide a useful tool for estimating possible changes in the drug metabolizing capacity of the liver in vivo. The intracellular metabolism in PBL may be an additional factor determining the concentration of drugs at their target site. Thus, the lymphocyte cytochrome P450 profile may be utilized as an indicator of the hepatic cytochrome P450 profile in further (clinical) studies with lurasidone, and it may serve as an easily available surrogate for exploring the impact of new drugs and long-term treatments on CYP enzyme expression and systemic metabolism. The lymphocyte cytochrome P450 system seems to be a reasonable biomarker for monitoring in vivo exposure to xenobiotics and for estimating the possibility of drug–drug interactions and toxicity risk during continuous exposure. However, the translation of results obtained using PBL from animals to humans requires further study.

## Figures and Tables

**Table 1:** Summary of changes in the expression and activity of cytochrome P450 enzymes in the liver and peripheral blood lymphocytes after chronic treatment of rats with lurasidone.

Cytochrome P450		CYP1A2	CYP2A1	CYP2A2	CYP2B1	CYP2B2	CYP2C11	CYP2E1	CYP3A1	CYP3A2
LIVER	Activity	No change	No change		↓		↓	↓	↑	
	Protein	No change	No change		↓		↓	↓	↑	↑
	mRNA	No change	No change	No change	↓	No change	↓	↓	↑	↑
LYMPHOCYTES	Protein	No change	No change		↓		↓	(↓)	↓	↑
	mRNA	No change	No change	No change	↓	↓	No change	↓	No change	↑

↓ decrease, (↓) tendency to decrease, ↑ increase.

**Table 2:** Protocol for Western Blot analysis.

CYP Enzymes	Microsomes			Lymphocytes		
	Primary Antibody	Secondary Antibody	Positive Control [μg]	Primary Antibody	Secondary Antibody	Positive Control [μg]
CYP1A	1:1000	1:4000	5	1:1000	1:1000	1
CYP2A	1:2000	1:4000	5	1:2000	1:1000	1
CYP2B	1:1000	1:4000	5	1:1000	1:1000	1
CYP2C11	1:1000	1:4000	5	1:1000	1:1000	1
CYP2E1	1:2000	1:4000	2	1:2000	1:1000	0.4
CYP3A1	1:4000	1:4000	1	1:4000	1:1000	0.2
CYP3A2	1:4000	1:4000	1	1:4000	1:1000	0.2
β-actin	1:10,000	1:2000	-	1:20,000	1:2000	-

## Data Availability

Data are contained within the article.

## Supplementary Materials

The following supporting information can be downloaded at https://www.mdpi.com/article/10.3390/ijms242316796/s1.
