# Supplementary material for: The Novel Atypical Antipsychotic Lurasidone Affects Cytochrome P450 Expression in the Liver and Peripheral Blood Lymphocytes
Source: Int J Mol Sci. 2023 Nov 27;24(23):16796. doi: 10.3390/ijms242316796 (PMC10706667; doi:10.3390/ijms242316796)

CYP1A

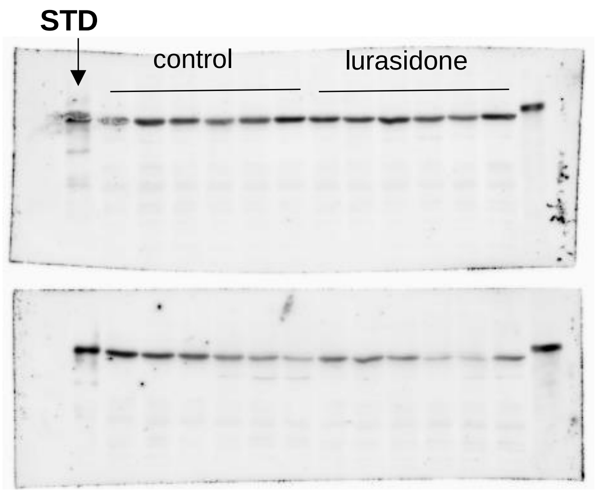

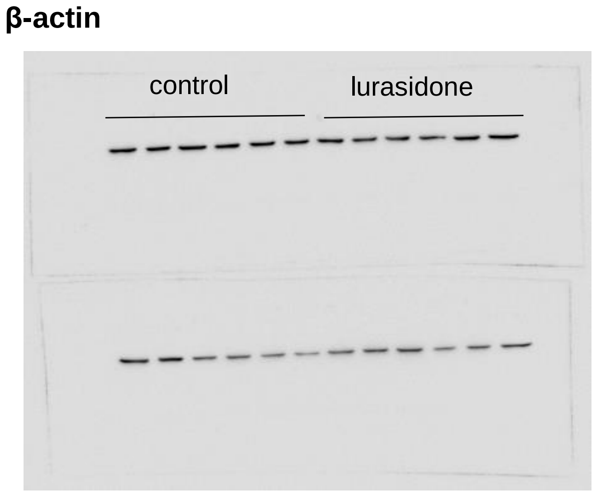

CYP2A

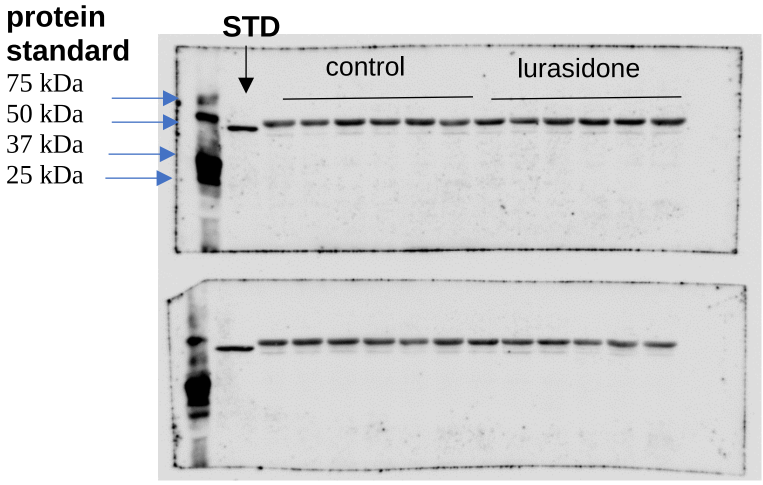

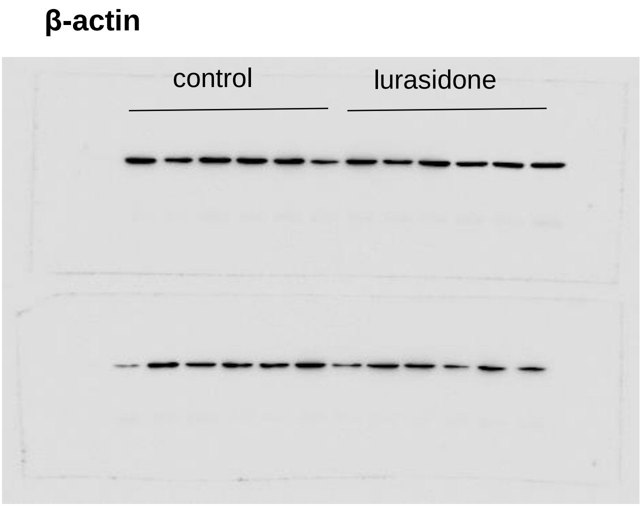

CYP2B

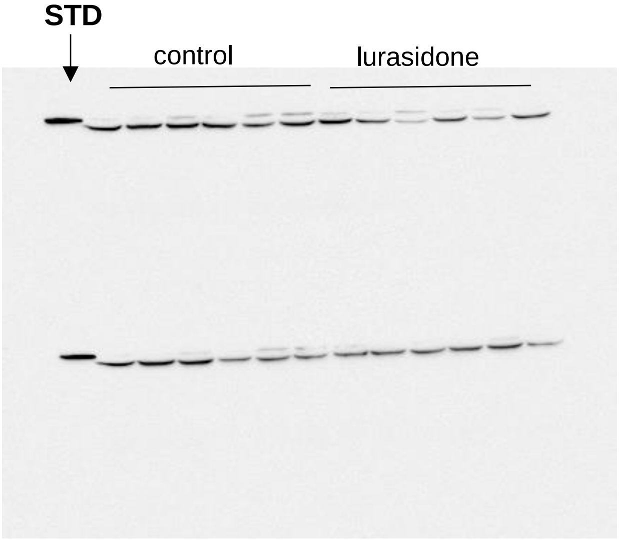

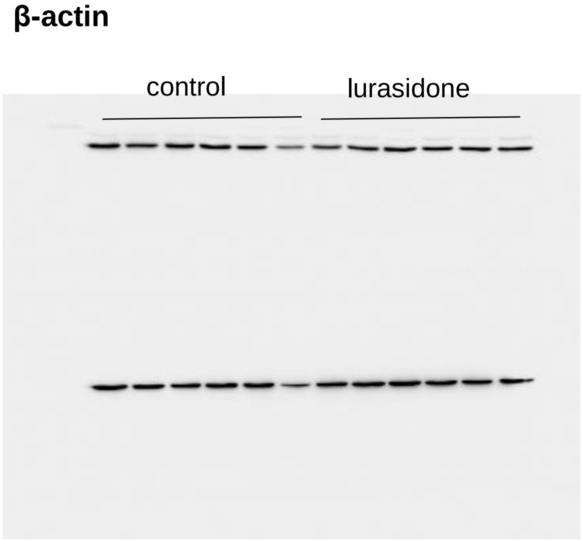

CYP2C11

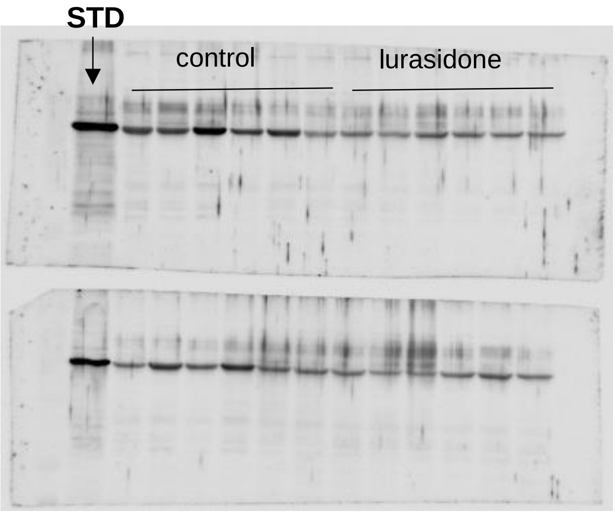

β-actin

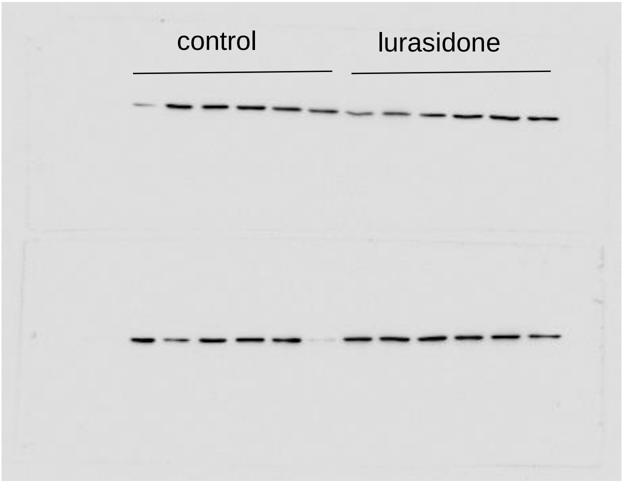

CYP2E1

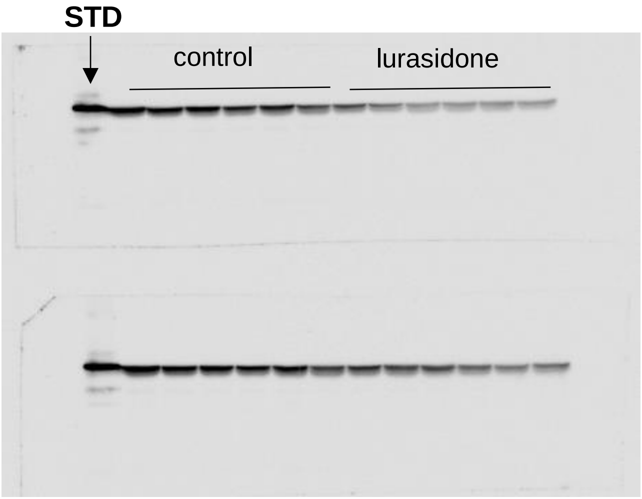

β-actin

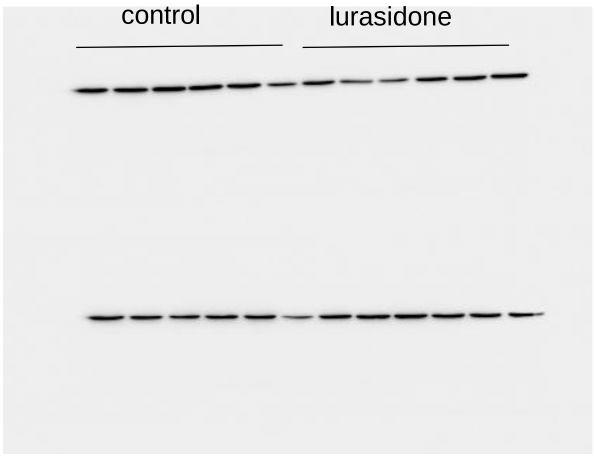

CYP3A1

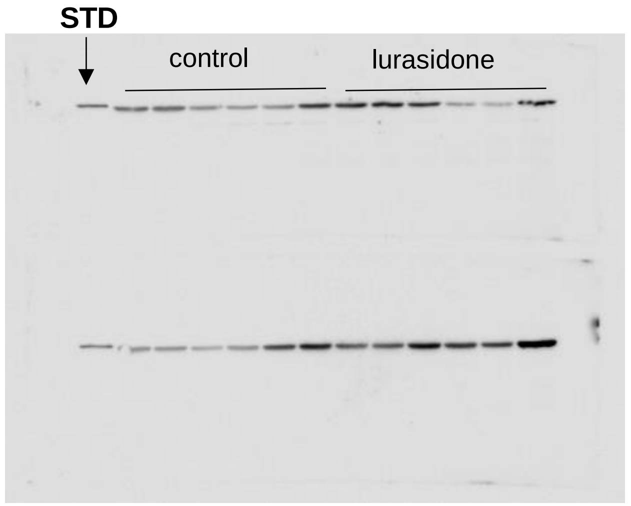

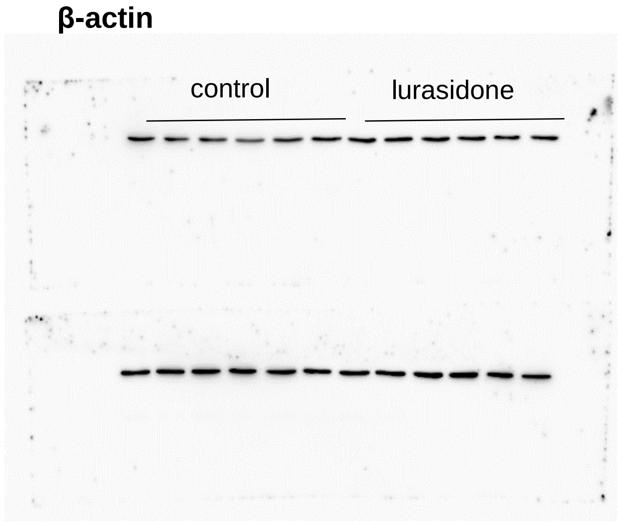

CYP3A2

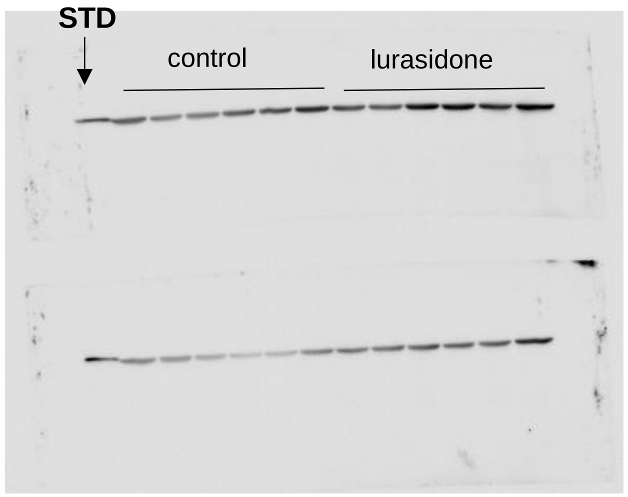

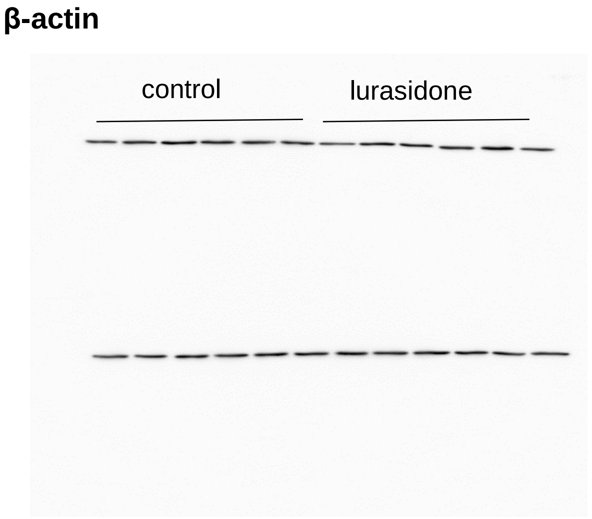

Supplement: Supplementary file 1 [file ijms-24-16796-s001.zip › ijms-2713213-supplementary.pdf]
